# Knowledge, attitude and practice towards tuberculosis among healthcare and non-healthcare students at a public university in Saudi Arabia

**DOI:** 10.3389/fpubh.2024.1348975

**Published:** 2024-02-05

**Authors:** Geetha Kandasamy, Dalia Almaghaslah, Mona Almanasef

**Affiliations:** Department of Clinical Pharmacy, College of Pharmacy, King Khalid University, Abha, Saudi Arabia

**Keywords:** tuberculosis, students, knowledge, attitude, practice, healthcare, non-healthcare

## Abstract

**Background:**

Tuberculosis (TB) is a bacterial infection. It mostly affects the lungs (pulmonary TB), but it can also affect other organs. This cross-sectional study evaluated knowledge, attitudes, and practices (KAP) related to TB among King Khalid University (KKU) students between October and November 2023.

**Objective:**

The objective of this study was to investigate current TB knowledge, attitudes, and practices of students at King Khalid University in Abha, Saudi Arabia.

**Methods:**

A self-administered, cross-sectional, descriptive, web-based questionnaire was conducted from October to December 2023 among the students of King Khalid University. We used a 29-item questionnaire with five sections. Section 1 contained five questions about sociodemographic factors, there were 13 knowledge questions in Section 2, Section 3 contained 7 attitude questions, Section 4 contained 3 practice questions, and Section 5 contained 1 source of information question. A chi-squared test was used to assess differences in participants’ knowledge, attitude, and practices in relation to their demographic variables (*p* < 0.05).

**Results:**

A total of 518 students completed the questionnaire. 53.66% were healthcare students and 46.33% non-healthcare students. The mean scores for healthcare and non-healthcare students, respectively, were as follows: knowledge 11.80 ± 4.81, 7.35 ± 4.96; attitude 6.94 ± 1.33, 5.05 ± 2.09; and practice 2.26 ± 0.85, 1.14 ± 0.87. The results of this study showed good knowledge (24.82 and 5.83% for healthcare and non-healthcare students, respectively) good attitude (67.62 and 46.25%) and good practice (45.32 and 9.58%). A total of 24.32% healthcare students and 28.18% non-healthcare students reported that most effective sources for obtaining information about TB were social networks, the internet and the radio.

**Conclusion:**

The current study concludes that the knowledge, attitude, and practice about TB among healthcare faculty students is better than their non-healthcare counterparts. However, there are still areas of poor knowledge, attitude and practice toward some aspects of TB among the two categories, which shows the necessity of educational intervention that aims at improving student understanding about the disease and its impact on public health.

## 1 Introduction

Aerosols expelled by patients with active tuberculosis (TB) disease can transmit the communicable disease tuberculosis, which is caused by the bacillus *Mycobacterium tuberculosis* (Mtb). The management and prevention of communicable diseases such as TB are highly dependent on the awareness of society and the healthcare professional network. Correct management of TB by trained healthcare professionals, early diagnosis, and community awareness are all potential approaches to solving this global health issue (1). According to the World Health Organization’s (WHO) Global Tuberculosis Report 2021, prior to the coronavirus (COVID-19) pandemic, TB was the 13th largest cause of death from a single infectious agent, over HIV/AIDS (2–4). Eradicating the TB pandemic by 2030 is one of the Sustainable Development Goals (SDGs) of the United Nations (3). Over a 20-year period, 64,345 cases were reported in Saudi Arabia; 48% of the cases were reported by people with non-Saudi nationalities, and Makkah had the highest incidence rate. During the Hajj season, around two million Muslims from over 180 nations go to Makkah to perform the Islamic pilgrimage, staying there for five to 6 days. This greatly contributes to the high occurrence rate. Furthermore, many tourists travel from regions where tuberculosis is prevalent to participate in rituals and worship under conditions that are believed to increase the risk of TB transmission. As a result, tuberculosis is quite prevalent during the Hajj season and is the most common cause of pneumonia that requires hospitalization (5). The perceived significance of addressing this burden is vastly different from the actual burden of the disease, even in areas where it is common (6). Aerosolized particles from coughing or sneezing are the main cause of Mtb infections. Males are affected more frequently than females and make up about 90% of those who contract the disease. Chest pain, weakness, weight loss, fever, and night sweats are just a few of the signs of pulmonary TB, a dangerous bacterial lung infection that spreads through the air when someone talks, sneezes, or coughs (7). The less well-known extrapulmonary infection sites are the skeletal and central nervous systems (8).

A total of 3,918 cases of tuberculosis were reported in Saudi Arabia in 2008, out of a total population of 24,807,273 people (9). Despite the fact that tuberculosis is a disease that can be prevented and treated, around 10 million people are diagnosed with it every year. In addition, TB kills 1.5 million people annually, making it a serious infectious illness (3). An estimated 10.6 million cases of tuberculosis were reported in 2021, and almost 1.6 million people died from the disease (3, 10).

Most of the people affected are young and middle-aged, with an average age between 15 and 44. The incidence of all cases was estimated to be 15.8 per 100,000 people, and the prevalence of all TB patients was projected to be 65 cases per 100,000 people annually (9). To reduce TB mortality and morbidity, public awareness is vital (11). It is well known that early detection of the disease and high public awareness are positively correlated. Knowledge has a significant impact on how people behave and go about their daily lives. The incidence and mortality of tuberculosis can be reduced with early identification and diagnosis (12). A more contemporary definition of health-related stigma is “a social process or related personal experience characterized by exclusion, rejection, blame, or devaluation that results from experience or reasonable anticipation of an unfavorable social judgment about an individual or group identified with a specific health problem” (13). World TB Day is held annually on March 24 in order to increase public awareness of tuberculosis (TB) and the terrible effects it has on people’s health, society, and economy, as well as to promote efforts to end the global TB pandemic (14).

Due to the presence of two sacred sites of worship in the Makkah and Al Madina districts, Saudi Arabia is one of the most important Muslim nations. Year-round pilgrimages by Muslims from throughout the world to the holy sites reach their zenith during the annual Hajj pilgrimage in the lunar month of Zul Hijah. Furthermore, the number of pilgrims for Umrah, a shorter pilgrimage carried out throughout the year, increases significantly during Ramadan (according to the lunar calendar). Infectious diseases, particularly respiratory tract infections, are more likely to spread in this environment (15). Young people aged 19 to 22 years are the age group for tuberculosis (TB), with most of these individuals being college or university students (16). Because of the dense population and high degree of interaction among individuals found in universities and schools, diseases like tuberculosis can spread more easily (17). Failure to seek medical attention is a very regular occurrence in this setting. Ignorance of preventative measures contributes to the failure to diagnose and cure tuberculosis in its early stages to some extent. Inadequate disease prevention strategies and inefficient utilization of medical resources are caused by low levels of awareness about tuberculosis. As a result, in order to identify miscommunication and identify the populations that would benefit from TB awareness raising programs, it is imperative that the level of current TB knowledge, attitudes, and practices (KAP) be assessed (18). Numerous global investigations examining university students’ KAP around TB have been conducted. For instance, a study on TB knowledge among 839 university students who were not medical professionals was carried out in Bangladesh. The researchers came to the conclusion that there is a lack of public knowledge about tuberculosis (19). Therefore, the purpose of this study was to explore current knowledge, attitude, and practices about tuberculosis among students at King Khalid University in Abha, Saudi Arabia. The results can be used to create successful TB control policies for the country, and will provide important insights into the current knowledge, attitude, and practices among students.

## 2 Materials and methods

### 2.1 Study design

A self-administered, cross-sectional, descriptive, web-based questionnaire was conducted from October to December 2023 among the students of King Khalid University.

### 2.2 Population criteria

All King Khalid University undergraduates, namely in the faculties of Dentistry, Pharmacy, Medicine, Nursing and Applied Health Sciences (Healthcare), as well as English, Mathematics, Engineering, and Computer Science (non-healthcare). They included those who are proficient in reading, writing and speaking English. Informed consent was obtained from the student volunteers who were willing to participate in the study after outlining the project goals. Students who did not provide their agreement to participate in the study, or produced incomplete questionnaires (13), were excluded.

### 2.3 Sample size and sampling procedure

One thousand and eighty-three students were approached, and 531 students responded. 518 completed the questionnaire completely, while the other 13 were excluded due to incomplete data. Study participants were chosen and enrolled using a snowball sampling technique.

### 2.4 Data collection tool

A standardized and verified questionnaire was used to collect information from study participants at the appropriate colleges. A Google Forms-created online survey was used for the study, and it was distributed to students between October and November 2023. The survey was distributed via social media, online forums, and other channels. The survey was completed willingly and anonymously by participants.

### 2.5 Questionnaire

We used a 29-item questionnaire with five sections based on a review of research that had similar objectives (15, 20–22). Five questions about sociodemographic factors, including age, gender, specialization, year of study, and smoking status were included in the first section of the questionnaire. There were 13 knowledge questions in Section 2, Section 3 contained 7 attitude questions, Section 4 contained 3 practice questions, and Section 5 contained 1 source of information question. Following Bloom’s cutoff point (23), students’ general knowledge, attitude, and practice were divided into three categories based on their total percentage score: good for scores of 80% and above, moderate for scores of 60–79%, and poor for scores of less than 60%. Participants were expected to respond “Yes,” “No,” or “Do not know” to the knowledge questions. Each accurate response received a score of 1, while “Do not know” received a score of 0. The maximum score for knowledge was 19, the maximum score for attitude was 10, and the range for practice questions was 1 to 3. Furthermore, we discovered a percentage of each response as the information source.

### 2.6 Statistical analysis

The Statistical Package of Social Sciences (SPSS) program, version 17, was used for the analysis. Descriptive statistics, including mean, standard deviation, frequency, and percentage were used to statistically analyze the collected data. Categorical variables and demographics were described using descriptive analysis, such as frequency and percentage. A Chi-square test was used to assess differences in participant knowledge, attitude, and practices in relation to demographic variables. The significance value was set at *p* < 0.05.

### 2.7 Ethics approval

This study, HAPO-06-B-001, was approved by King Khalid University Research Ethics Committee, with approval reference ECM 2023–3,113. Before participating in the study, all respondents were asked for their signed consent. It was completely the decision of participants to accept or decline the invitation to participate in the study.

## 3 Results

### 3.1 Sociodemographic characteristics

A total of 518 students completed the questionnaire: 53.66% (*n* = 278) healthcare students and 46.33% (*n* = 240) non-healthcare students. Among the healthcare participants, 42.08% (*n* = 117) were male and 47.91% (*n* = 115) were female, while 57.91% (*n* = 161) were male and 52.08% (*n* = 125) were female among the non-healthcare counterparts (*p* = 0.18). Smokers among the healthcare students represented 33.81% (*n* = 94), while this was 21.66% (*n* = 52) for non-healthcare students (*p* < 0.01). Detailed demographic information of the study participants is presented in Table 1.

**Table 1:** Demographic variables among healthcare and non-health care students of university.

Variables	Healthcare students (*n* = 278) %	Non-healthcare students (*n* = 240) %	Total (*n* = 518)	X^2^	*p* value
Gender				1.77	0.18
Male	117 (42.08%)	115 (47.91%)	232		
Female	161 (57.91%)	125 (52.08%)	286		
Smoking				9.38	*0.0021
Yes	94 (33.81%)	52 (21.66%)	146		
No	184 (66.18%)	188 (78.33%)	372		
Age in Years				16.79	*0.0002
18–20	112 (40.28%)	58 (11.19%)	170		
21–25	110 (39.56%)	132 (25.48%)	242		
>25 Years	56 (20.14%)	50 (9.65%)	106		
Year of Study				10.44	*0.033
1	21 (7.55%)	8 (3.33%)	29		
2	44 (15.82%)	25 (10.41%)	69		
3	77 (7.69%)	62 (25.83%)	139		
4	76 (27.33%)	85 (35.41%)	161		
5	60 (21.58%)	60 (25%)	120		

X^2^, Chi Square test, Significance *p* < 0.05.

### 3.2 Students’ knowledge of tuberculosis

The study findings showed that among the healthcare and non-healthcare students, respectively, 24.82% (*n* = 69) and 5.83% (*n* = 14) had good knowledge about TB, 44.60% (*n* = 124) and 25.41% (*n* = 61) had moderate knowledge, and 30.57% (*n* = 85) and 68.75% (*n* = 165) had overall poor knowledge of TB, with the mean (± SD) scores for knowledge being 11.80 ± 4.81 and 7.35 ± 4.96 (Table 2).

**Table 2:** Scores of KAP among the healthcare and nonhealthcare students of university.

Scores	Healthcare (*n* = 278)			Non-healthcare (*n* = 240)		
	Good (80%)	Moderate (60–79%)	Poor (Less than 60%)	Good (80%)	Moderate (60–79%)	Poor (Less than 60%)
Knowledge	69 (24.82)	124 (44.60)	85 (30.57)	14 (5.83)	61 (25.41)	165 (68.75)
Attitude	80 (28.77)	182(65.46)	16 (5.75)	30 (12.50)	76 (31.66)	134 (55.83)
Practice	126 (45.32)	89 (32.01)	63 (22.66)	23 (9.58)	39 (16.25)	178 (74.16)

In the current study, 74.46% of healthcare students and 46.25% of the non-healthcare students had heard of tuberculosis. 40.28% of healthcare students and 13.75% of non-healthcare students believed that TB can occur anywhere in the body. A total of 72.66% healthcare students and 36.25% non-healthcare students confirmed that smoking causes TB. Only 73.74% (healthcare) and 46.66% (non-healthcare) students confirmed that cold air cause TB; for and dust, the proportions were 73.38 and 41.25%. A total of 72.30% healthcare students and 47.5% non-healthcare students knew that TB can be transmitted by coughing/breathing. A total of 14.38% of healthcare students reported that TB can be transmitted through sexual contact, compared to 27.33% of non-healthcare students. Knowledge of symptoms of TB from the participants are as follows: cough for 2 weeks, 74.46% (healthcare) and 38.33% (non-healthcare students); chest pain, 68.70% (healthcare) and 34.58% (non-healthcare); weight and appetite loss, 61.15% (healthcare) and 37.91% (non-healthcare); and night sweating, 66.90% (healthcare) and 30.83% (non-healthcare). Only 42.44% of healthcare students reported that TB is a preventable disease, compared to 31.25% of non-healthcare students. A total of 47.84% healthcare students reported that TB can be prevented by covering the mouth while coughing compared to 26.25% non-healthcare students. Only 51.79% healthcare students and 27.5% non-healthcare students indicated that TB can be cured. A total of 16.54% healthcare students and 36.66% non-healthcare students said that TB can be cured by herbal remedies. A total of 11.87% healthcare students and 17.91% non-healthcare students reported that TB can be cured by home rest without treatment. What is more, 16.18% of students from healthcare and 48% from non-healthcare faculties indicated that TB can only be treated if there are obvious symptoms. Only 62.58% (healthcare) and 10.43% (non-healthcare) of students had knowledge that TB treatment is difficult and that if anti-TB drugs are not taken regularly, it can lead to drug resistance. Only 80.21% of students from healthcare were aware that TB treatment is free in Saudi Arabia. Additional data on students’ knowledge are presented in Table 3.

**Table 3:** Knowledge toward tuberculosis among healthcare and non-health care students of university.

Questions	Healthcare students (*n* = 278) %			Non-healthcare students (*n* = 240) %		
	Yes n (%)	No n (%)	Do not know n (%)	Yes n (%)	No n (%)	Do not know n (%)
1. Have you ever heard of an illness called TB?	207 (74.46)	24 (8.63)	47(16.90)	111 (46.25)	75 (31.25)	54 (22.5)
2. TB can occur anywhere in the body.	112 (40.28)	95 (34.17)	71 (25.53)	33 (13.75)	145 (60.41)	62 (25.83)
3. What causes Tuberculosis?						
a. Cold Air	205 (73.74)	53 (19.06)	20 (7.19)	112 (46.66)	79 (46.66)	49 (20.41)
b. Smoking	202 (72.66)	40 (14.38)	36 (12.94)	87 (36.25)	78 (32.5)	75 (31.25)
c. Dust	204 (73.38)	49 (17.62)	25 (8.99)	99 (41.25)	86 (35.83)	55 (22.91)
4. Can TB transmit through…?						
a. Cough/breathe	201 (72.30)	43 (15.46)	34 (12.23)	114 (47.5)	96 (40.00)	30 (12.50)
b. Sexual contact	40 (14.38)	213 (76.61)	25 (8.99)	76 (27.33)	119 (49.58)	45 (18.75)
5. What are Symptoms of Tuberculosis?						
a. Cough for two weeks	207 (74.46)	25 (8.99)	46 (16.54)	92 (38.33)	85 (35.41)	63 (26.25)
b. Chest pain	191 (68.70)	43 (15.46)	44 (15.82)	83 (34.58)	95 (39.58)	62 (25.83)
c. Weight and appetite loss	170 (61.15)	55 (19.78)	53 (19.06)	91 (37.91)	83(34.58)	66 (27.50)
d. Night sweating	186 (66.90)	60 (21, 0.58)	32 (11.51)	74 (30.83)	93 (38.75)	73 (30.41)
6. Is TB preventable disease?	118 (42.44)	97 (34.89)	63 (22.66)	75 (31.25)	67 (27.91)	98 (40.83)
7. Can TB be prevented by covering mouth while coughing?	133 (47.84)	66 (23.74)	79 (28.41)	63 (26.25)	85 (35.41)	92 (38.33)
8. Can TB be Cured?	144 (51.79)	95 (34.17)	39 (14.02)	66 (27.5)	77 (32.08)	97 (40.41)
9. Can TB be cured by herbal remedies?	46 (16.54)	183 (65.82)	49 (17.62)	88 (36.66)	125 (52.08)	27 (11.25)
10. Can TB be cured by home rest without treatment?	33 (11.87)	176 (63.30)	69 (24.82)	43 (17.91)	101 (42.08)	96 (40.00)
11. TB can only be treated if there are obvious symptoms.	45 (16.18)	159 (57.19)	74 (26.61)	48 (20.00)	138 (57.50)	54 (22.50)
12. Is TB treatment difficult and if anti TB drugs are not taken regularly, it can lead to drug resistance?	174 (62.58)	75 (26.97)	29 (10.43)	87 (36.25)	119 (49.58)	38 (15.83)
13. Is TB treatment free in Saudi Arabia?	223 (80.21)	30 (10.79)	15 (5.39)	95 (39.58)	86 (35.83)	59 (24.58)

### 3.3 Attitude toward tuberculosis among healthcare and non-healthcare students

The results of this study showed that among the healthcare and non-healthcare students, respectively, 28.77% (*n* = 80) and 12.50% (*n* = 30) had a good attitude toward TB, 65.46% (*n* = 182) and 31.66% (*n* = 76) had a moderate attitude, while 5.75% (*n* = 16) and 55.83% (*n* = 134) had a poor attitude. The mean (± SD) scores for attitude were 6.94 ± 1.33 and 5.05 ± 2.09 for healthcare and non-healthcare students, respectively (Table 2).

Only 67.62% of students in healthcare are willing to work with someone previously treated for TB, whereas 46.25% of students from non-healthcare are. A total of 15.10% of students in healthcare want TB in a family member to be kept secret, while this is true for 30.41% of non-healthcare students. Only 14.74% (healthcare) and 34.16% (non-healthcare) students believed that they can get TB. The reaction of participants if they were found to have TB would be: fear, 8.27% (healthcare) and 22.91% (non-healthcare); hopelessness, 6.47 and 12.5%; shame, 15.10 and 10.41%; sadness, 14.38 and 22.91%; and acceptance, 55.75 and 31.25%. In terms of having interest in finding out more about TB, only 64.38 and 34.58% of students strongly agree, 32.01 and 42.5% agree, and 3.59 and 22.91% disagree. Only 53.23% (healthcare) and 31.66% (non-healthcare) strongly agree, 41.36 and 42.5% agree, and 5.39 and 25.83% disagree that education about TB is very much needed. 66.54% (healthcare) and 38.75% (non-healthcare) strongly agree, 27.33 and 35.41% agree, and 6.11 and 25.83% disagree that they should encourage those around them with TB to obtain treatment (Table 4).

**Table 4:** Attitude toward tuberculosis among healthcare and non-health care students of university.

Response	Healthcare students (*n* = 278)			Non-healthcare students (*n* = 240)		
	Yes n (%)	No n (%)	Do Not Know n (%)	Yes n (%)	No n (%)	Do Not Know n (%)
Would you be willing to work with someone previously treated for TB?	188 (67.62)	35 (12.58)	55 (19.78)	111 (46.25)	23 (9.58)	106 (44.16)
Would you want a family member’s TB to be kept secret?	42 (15.10)	139 (50.00)	97 (34.89)	73 (30.41)	116 (48.33)	51 (21.25)
Do you think you can get TB?	41 (14.74)	135 (48.56)	102 (36.69)	82 (34.16)	117 (48.75)	41 (17.08)
What would be your reaction if you found out that you have TB?						
Response	Fear		Hopelessness	Shame	Sadness	Acceptance
Healthcare students (*n* = 278)	23 (8.27)		18 (6.47)	42 (15.10)	40 (14.38)	155 (55.75)
Non-healthcare students (*n* = 240)	55 (22.91)		30 (12.50)	25 (10.41)	55 (22.91)	75 (31.25)
I am interested in finding out more about TB.						
Response	Strongly Agree		Agree	Disagree		
Healthcare students (*n* = 278)	179 (64.38)		89 (32.01)	10 (3.59)		
Non-healthcare students (*n* = 240)	83 (34.58)		102 (42.5)	55 (22.91)		
I think education about TB is very much needed						
Response	Strongly Agree		Agree	Disagree		
Healthcare students (*n* = 278)	148 (53.23)		115 (41.36)	15 (5.39)		
Non-Healthcare students (*n* = 240)	76 (31.66)		102 (42.50)	62 (25.83)		
I would encourage those with TB around me to obtain treatment						
Response	Strongly Agree		Agree	Disagree		
Healthcare students (*n* = 278)	185 (66.54)		76 (27.33)	17 (6.11)		
Non-healthcare students (*n* = 240)	93 (38.75)		85 (35.41)	62 (25.83)		

### 3.4 Practice toward tuberculosis among healthcare and non-healthcare students

The results of this study showed that among the healthcare and non-healthcare students, respectively, 45.32% (*n* = 126) and 9.58%% (*n* = 23) had good practice toward TB, 32.01% (*n* = 89) and 16.25%% (*n* = 39) had moderate practice, while 22.66% (*n* = 63) and 74.16% (*n* = 178) had poor practice. The mean (± SD) scores for practice were 2.26 ± 0.85 and 1.14 ± 0.87 for healthcare and non-healthcare students, respectively (Table 2).
  Among the healthcare and non-healthcare students, respectively, only 76.97 and 30.41% would go to a health facility if they had symptoms of TB, 5.39 and 20.41% would go to a pharmacy, 8.99 and 36.66% would go to traditional healers, and 8.63 and 12.5% would seek self-treatment. Among the healthcare students, 76.61% reported that they would seek medical help as soon as they realized they had TB symptoms. Among the non-healthcare students, this number was 52.5%.
  Reasons reported by students for not going to a healthcare facility were as follow: 2.87% (healthcare) and 18.75% (non-healthcare) indicated that they were not sure where to go, 13.30 and 27.08% said it was due to cost of the services, 2.15 and 10.83% do not trust healthcare workers, and 8.99 and 12.5% of students said that they could not leave their work (Table 5).
  Participants from healthcare and non-healthcare disciplines in this study reported that the most effective sources for obtaining information about TB were newspapers and magazines (24.32 and 18.14%), social media, internet and radio (24.32 and 28.18%), television (10.42 and 10.8%), brochures, posters, and other printed materials (18.52 and 13.5%), health workers (10.8 and 8.88%), family, friends, neighbors, and colleagues (13.88 and 8.48%), and teachers (2.50 and 2.31%).

**Table 5:** Practice toward tuberculosis among healthcare and non-health care students of university.

1. What would you do if you thought you had symptoms of TB?					
Response	Go to a health facility	Go to a pharmacy	Go to traditional healers	Self-treatment
Healthcare students (*n* = 278)	214 (76.97)	15 (5.39)	25 (8.99)	24 (8.63)
Non-healthcare students (*n* = 240)	73 (30.41)	49 (20.41)	88 (36.66)	30 (12.50)
2. If you had symptoms of TB, at what point would you seek medical help?					
	When treatment on my own does not work	When TB symptoms last for two or more weeks	As soon as I realize TB symptoms	Do not know
Healthcare students (*n* = 278)	16 (5.75)	26 (9.35)	213 (76.61)	23 (8.27)
Non-healthcare students (*n* = 240)	35 (14.58)	64 (26.66)	126 (52.5)	15 (6.25)
3. If you would not go to the health facility, what is the reason?					
Response	I did not refuse to go to the hospital	Not sure where to go	Cost of the services	Do not trust health care workers	Cannot leave my work
Healthcare students (*n* = 278)	202 (72.66)	8 (2.87)	37 (13.30)	6 (2.15)	25(8.99)
Non-healthcare students (*n* = 240)	74 (30.83)	45 (18.75)	65 (27.08)	26 (10.83)	30 (12.50)

## 4 Discussion

The current study reveals that healthcare students exhibited better knowledge about TB than non-healthcare students. They were more aware of the disease’s cause, symptoms, transmission, prevention, prognosis and treatment. However, around one quarter of healthcare faculty students and only 5.8% of non-healthcare faculty students showed good knowledge about the disease. Similar findings were reported in an Ethiopian study among the same population, where 35% were healthcare faculty students (24). Another study conducted in Afghanistan concluded similar results, with less than a quarter of healthcare faculty students and 2.4% of non-healthcare faculty students having good knowledge (22). An Indonesian study revealed that healthcare students obtained higher scores in knowledge assessment with an average of 7.03 ± 2.36 out of 11 compared to non-healthcare students who scored 4.98 ± 2.36 out of 11 on average (18). A Chinese study indicated a low awareness level of TB among freshmen university students enrolled in various colleges (17). On the other hand, a study conducted in Iran indicated moderate to high knowledge among healthcare students with an average score of 16.3 ± 2.06 (low <10, moderate 10–15, high >15) (25, 26). A recent literature review has found that non-healthcare students in Sweden, Serbia, and Bangladesh had poor knowledge about TB as well as some misconceptions. It also reported that healthcare students in Rome, Brazil, Nigeria, and China had poor knowledge about the disease (27). Similar findings were reported in a study carried out in India, where healthcare students’ knowledge was assessed through a pre-test questionnaire, and their average score was 48.59 (± 20.44) (28). In Jordan, students reported knowledge gaps in TB treatment and transmission with a median score of 27 out of 51 (29).

A national study conducted in Saudi Arabia revealed poor overall knowledge among students with grades of 51.4% (30). However, healthcare students obtained higher grades (46.7%) in comparison to non-healthcare students (27.2%). The level of students’ knowledge in the current study and in previous studies indicate that there should be more focus on communicable diseases such as TB, especially in countries where the incidence of the disease is high. Healthcare disciplines curricula should be tailored to widen students’ knowledge on all aspects of the disease. For example, around a quarter of healthcare students and more than half of non-healthcare students were not aware of extrapulmonary TB. Another example is the misconception students have about the causes of the disease, with around a quarter of healthcare students believing that cold weather and dust are the causes of TB. Hence, healthcare curricula should include the modules that covers TB disease’s cause, symptoms, transmission, prevention, prognosis and treatment according to the specialty. On the other hand, non-healthcare students should be educated through health campaigns and awareness programs (18). Students at the medical colleges were more likely to have good/excellent TB-related knowledge and attitudes (*p* < 0.001 each) when compared with their peers in non-medical colleges (31).

Just over two thirds of healthcare students and under half of non-healthcare students had positive attitudes toward TB. A previous study conducted in Afghanistan showed that healthcare students had better attitudes than non-healthcare students (22). Similar findings were reported in Iran, where most healthcare students had moderate to good attitudes toward TB (25). Another investigation in Iraq reported having around the same proportion of healthcare students (76%) who had positive attitudes toward TB (16). Non-healthcare students’ attitudes to tuberculosis are negative, according to a Swedish study (32). Another study in Rome reported a moderate level of general knowledge about tuberculosis, which shows the need to adapt present programs of infectious diseases in the curriculum of medical schools (33). The results of two other investigations (26, 34) supported our findings that medical students have a favorable attitude toward tuberculosis. This discrepancy might result from medical colleges’ official teaching of tuberculosis in their undergraduate curricula, which are incorporated into the internship program at each health college. In the realm of infectious illnesses, health college students receive formal instruction on health themes, specifically tuberculosis. Because of this, health college students are more knowledgeable, more conscious, and have more favorable attitudes and behaviors toward the prevention of infectious diseases like TB (31). Another investigation that evaluated KAP among the Saudi general public revealed that the majority of participants had a negative attitude toward tuberculosis (34).

Assessing students’ practices toward TB showed that both healthcare students and non-healthcare students would seek care from healthcare facilities, indicating some level of awareness, but some would seek help from traditional healers, which is alarming behavior, especially for healthcare students. The reasons for not seeking professional medical help from a healthcare facility varied, with not wanting to go a hospital being the top reason. Other reasons included not being sure where to go, and the cost of the treatment. Hence, students should be educated and further encouraged to seek healthcare from professional bodies (25). When it comes to the general population outside of universities, in a study in the Makkah region, Saudi Arabia, 89.9% of the respondents demonstrated poor attitudes, whereas only 2.3% had good attitudes (5).

While 61.4% of participants in the Saudi Arabian study reported that they would visit a doctor if they experienced TB symptoms, 76.97% of participants in this study reported that they would visit a health center (5). While 24.32% (healthcare) and 28.18% (non-healthcare) students in this survey indicated that their source of information was the internet and social media, the majority of participants in a Malaysian study of students in healthcare and non-healthcare faculties claimed that their source of Information was the internet (24).

Some of the attitudes that healthcare students have toward TB such as willingness to work with someone who was previously treated for TB and wishing to keep the disease a secret might negatively impact care-seeking behaviors of patients, due to possible stigma and discrimination. Hence, healthcare students’ misconceptions necessitate raising their awareness, accountability, and responsibility toward their professional role as healthcare personnel in the future (35). Newspapers, magazines, and social media were the main sources of information about TB. Therefore, educational campaigns can be recommended as the most suitable medium for future campaigns, considering that information is tailored to viewers of all health literacy levels (24).

The results of this study indicate that more public awareness efforts are required to inform people about tuberculosis and its symptoms, transmission, and treatment. Numerous strategies, including the media, community outreach initiatives, and health education seminars, could be used to accomplish this. The results of the study and the lack of knowledge, attitudes, and practices regarding tuberculosis among students indicate that TB education can be incorporated into the curricula of both healthcare and non-healthcare faculty members. Widening students’ knowledge on infection control as well as improving their attitudes and practices is essential for protecting healthcare students when performing patient care. This can aid student” comprehension of tuberculosis and its adverse impacts on public health.

It is important to note that the findings of this study could have been impacted by recollection bias. Since the study only involved one university, it is not possible to generalize the results to the entire country. This was one of the major limitations of our study. Future national studies should involve more universities with a large representative sample of the Saudi student population to have a better understanding of the scope of the problem.

## 5 Conclusion

The current study concludes that the knowledge, attitude, and practice about TB among healthcare faculty students is better than their non-healthcare counterparts. However, there are still areas of poor knowledge, attitude and practice toward some aspects of TB among the two categories, which shows the necessity of educational interventions that aim at improving students’ understanding about the disease and its impact on public health.

## Data availability statement

The raw data supporting the conclusions of this article will be made available by the authors, without undue reservation.

## Ethics statement

The studies involving humans were approved by the Research Ethics Committee at King Khalid University (HAPO-06-B-001) ECM 2023–3 113. The studies were conducted in accordance with the local legislation and institutional requirements. The participants provided their written informed consent to participate in this study.

## Author contributions

GK: Conceptualization, Data curation, Methodology, Resources, Validation, Writing – original draft, Writing – review & editing. DA: Conceptualization, Data curation, Formal analysis, Funding acquisition, Investigation, Resources, Software, Supervision, Validation, Writing – review & editing. MA: Conceptualization, Data curation, Funding acquisition, Methodology, Project administration, Software, Supervision, Validation, Visualization, Writing – original draft, Writing – review & editing.
